# Exploring the Biologically Active Metabolites Produced by *Bacillus cereus* for Plant Growth Promotion, Heat Stress Tolerance, and Resistance to Bacterial Soft Rot in *Arabidopsis*

**DOI:** 10.3390/metabo13050676

**Published:** 2023-05-22

**Authors:** Sih-Huei Tsai, Yi-Chun Hsiao, Peter E. Chang, Chen-En Kuo, Mei-Chun Lai, Huey-wen Chuang

**Affiliations:** Department of Bioagricultural Sciences, National Chiayi University, Chiayi 600355, Taiwan

**Keywords:** de novo whole-genome assembly, RNA-seq analysis, volatile organic compounds, serine proteases

## Abstract

Eight gene clusters responsible for synthesizing bioactive metabolites associated with plant growth promotion were identified in the *Bacillus cereus* strain D1 (BcD1) genome using the de novo whole-genome assembly method. The two largest gene clusters were responsible for synthesizing volatile organic compounds (VOCs) and encoding extracellular serine proteases. The treatment with BcD1 resulted in an increase in leaf chlorophyll content, plant size, and fresh weight in *Arabidopsis* seedlings. The BcD1-treated seedlings also accumulated higher levels of lignin and secondary metabolites including glucosinolates, triterpenoids, flavonoids, and phenolic compounds. Antioxidant enzyme activity and DPPH radical scavenging activity were also found to be higher in the treated seedlings as compared with the control. Seedlings pretreated with BcD1 exhibited increased tolerance to heat stress and reduced disease incidence of bacterial soft rot. RNA-seq analysis showed that BcD1 treatment activated *Arabidopsis* genes for diverse metabolite synthesis, including lignin and glucosinolates, and pathogenesis-related proteins such as serine protease inhibitors and defensin/PDF family proteins. The genes responsible for synthesizing indole acetic acid (IAA), abscisic acid (ABA), and jasmonic acid (JA) were expressed at higher levels, along with *WRKY* transcription factors involved in stress regulation and *MYB54* for secondary cell wall synthesis. This study found that BcD1, a rhizobacterium producing VOCs and serine proteases, is capable of triggering the synthesis of diverse secondary metabolites and antioxidant enzymes in plants as a defense strategy against heat stress and pathogen attack.

## 1. Introduction

In a natural environment, the rhizosphere is greatly populated by diverse microorganisms that show complex interactions with plant root systems. Plant growth-promoting rhizobacteria (PGPR) are soil microorganisms that colonize plant roots and exhibit potential functions in improving plant growth and stress tolerance. These PGPR produce a wide range of secondary metabolites, such as phytohormones, biofilm constituents, and siderophores, which not only serve as adaptation strategies for defense against various biotic and abiotic stresses, but also as beneficial elicitors for plant growth and development [1]. Among these microbial metabolites, indole acetic acid (IAA), the most abundant phytohormones of the auxin class, functions as a signaling molecule that regulates gene expression associated with the interactions between microbes and hostplants [2]. The IAA produced by microorganisms exhibits bioactivity in promoting plant growth due to its ability to alter plant root architecture and enhance nutrient uptake efficiency in colonized plants [3]. The extracellular exopolysaccharide (EPS) is a constituent of biofilms that facilitate bacterial colonization in plant roots. PGPR that produce EPS exhibit beneficial effects on plants by enhancing soil moisture in water-deficit conditions and mitigating damage caused by drought stress [4]. Plants treated with microbial EPS increase their content of osmolytes, such as proline and sugars, which strengthen their tolerance to drought stress [5]. Additionally, EPS-producing PGPR can improve salt stress tolerance in colonized plants by chelating Na^+^ ions and reducing Na^+^ uptake by plant roots [6]. A PGPR strain stimulates plant growth and increase tolerance to drought stress by producing polyamines, which are another constituent of biofilm [7]. Siderophores are organic compounds with low molecular weight that regulate iron availability for microbial and plant cells by chelating ferric iron from the environment [8]. Siderophores also exhibit antibiotic activity due to their role in regulating the availability of iron, an essential micronutrient for all organisms [9].

Rhizobacteria produce various types of metabolites with antibiotic activity that serve as weapons to inhibit the growth of their competitors [10]. Due to their ability to effectively control plant diseases, the antibiotic metabolites produced by PGPR are a significant contributor to the promotion of plant growth. For example, PGPR strains producing diacetylphloroglucinol (DAPG) and phenazine can assist or promote growth and development of a plant by inhibiting pathogen growth and inducing defense responses in plants [11,12]. Volatile organic compounds (VOCs) produced by rhizobacteria, such as 2,3-butanediol and dimethyl disulfide, have antimicrobial properties [13,14]. The production of VOCs by PGPR strains induces a systemic response that fortifies plants against abiotic stress and pathogen attack [15,16]. Beneficial effects of rhizobacteria on plant growth can also be achieved by PGPR, which can increase the availability of phosphate in the soil [17]. The enzyme 1-aminocyclopropane-1-carboxylate (ACC) deaminase produced by rhizobacteria converts ACC to ammonia and α-ketobutyrate, which can be used as a nutrient source for bacterial growth [18]. PGPR producing ACC deaminase can alleviate the growth inhibition effect mediated by ethylene under stressful conditions [19]. Rhizobacteria produce hydrolytic enzymes, including proteases and chitinases, that function as antifungal agents and help to control plant disease resistance [20,21]. An increased resistance against blast disease in rice plants was observed through the application of protease-secreting PGPR. This extracellular protease serves as one of the compounds that determine their biocontrol activity [22].

Jasmonic acid (JA) and salicylic acid (SA) are two phytohormones playing crucial roles in regulating plant resistance to biotic stress [23]. These two phytohormones transcriptionally activate genes encoding specific sets of pathogenesis-related (PR) proteins to enhance plant resistance against various pathogens. In *Arabidopsis*, SA induces the expression of genes encoding PR-1, PR-2, and PR-5, which leads to systemic acquired resistance (SAR) and protects plants against biotrophic pathogens [24]. In contrast, JA induces the expression of genes encoding PR-3, PR-4, and PR-12, resulting in induced systemic resistance (ISR) that protects plants against necrotrophic pathogens [24]. Plant secondary metabolites, such as glucosinolates, alkaloids, and terpenoids, play a role in plant defense response against pathogen and pest attack [25,26]. However, the JA signal is a positive regulator for glucosinolates and terpenoids accumulation in plants [27,28]. Various rhizobacteria show their potential in controlling plant disease by producing effective metabolites, which can manipulate plant signaling pathways linked to disease resistance in plants. For example, dimethyl disulfide can stimulate systemic defense against pathogens through the activation of the SA signaling pathway in plants [14]. Another VOC, 2,3-butanediol, elicits disease resistance in pepper plants against multiple viruses by activating both the SA and JA signaling pathways [15]. Rhamnolipid, a biosurfactant compound produced by certain rhizobacteria, can induce disease resistance against biotrophic, hemibiotrophic, and necrotrophic pathogens by activating the SA signaling pathway in *Arabidopsis thaliana* (ecotype Col-0) plants [29]. A rhamnolipid-producing strain of *Pseudomonas aeruginosa* induced disease resistance against *Fusarium oxysporum* f.sp. *cubense* Tropical Race 4 (*Foc* TR4) by activating the JA signal in banana plants [30].

In a variable abiotic stress environment, dysfunctional metabolic processes can lead to the accumulation of reactive oxygen species (ROS), which can cause oxidative damage to cellular molecules like proteins, lipids, and nucleic acids within cellular compartments [31]. The antioxidant defense system, which helps plants alleviate oxidative stress, relies on the ascorbate-glutathione cycle comprising several enzymes such as ascorbate peroxidase (APX), monodehydroascorbate reductase (MDAR), dehydroascorbate reductase (DHAR), and glutathione reductase (GR) [32]. Moreover, in response to stressful conditions, plant cells produce various secondary metabolites that exhibit antioxidant activity, thereby enabling them to counteract the buildup of oxidative stress. For example, isoprenoids and phenylpropanoids are two such metabolites that offer antioxidant properties to alleviate oxidative stress caused by excessive light exposure [33]. Flavonoids, a class of phenolic compounds present in plant tissues, account for the majority of antioxidant activity in plants [34]. Flavonoid accumulation has been documented in plants subjected to a variety of stressors, such as high temperatures, intense light, and drought [35]. In addition to their regulatory roles in the induced immunity response, both the SA and JA signaling pathways are involved in activating the antioxidant defense system to facilitate physiological adjustments in plants for developing abiotic stress tolerance [36,37]. The phytohormone ABA is a crucial regulator of the adaptation response to osmotic stress [38]. It has been found that ABA-induced tolerance to drought stress is correlated with an increase in antioxidant activity in plants [39]. Rhizobacteria can activate the antioxidant defense system to reduce oxidative damage caused by abiotic stress. For example, by enhancing the activity of antioxidant enzymes, including APX, guaiacol peroxidase (POD), and catalase, the application of *Bacillus firmus* treatment improved the salt stress tolerance of soybean plants [40]. Plant seedlings that received treatment from *Bacillus licheniformis*, containing genes responsible for producing 2,3-butanediol, demonstrated enhanced resilience against heat and drought stresses. Additionally, the treatment induced the activation of genes associated with the JA and ABA signaling pathways and involved in the production of antioxidants [41]. Treating plants with *Bacillus mycoides*, which produced metabolites with potent radical scavenging activity, resulted in the activation of antioxidant enzymes and increased accumulation of secondary metabolites with ROS scavenging activity. The treated plants demonstrated enhanced tolerance to abiotic stress, along with the upregulation of gene expression associated with the SA and JA signaling pathways [42].

The objective of this study was to conduct a genome-wide analysis of gene clusters linked to the synthesis of metabolites that promote plant growth in a rhizobacterial strain referred to as BcD1. Eight gene clusters were identified that are involved in this process, with the two largest groups being genes responsible for synthesizing VOCs and encoding serine proteases. BcD1 treatment resulted in increased accumulation of lignin and secondary metabolites, as well as enhanced activity of antioxidant enzymes. Additionally, the treatment promoted plant growth and increased stress tolerance against both abiotic and biotic stress. Transcriptome analysis showed that BcD1 treatment activated genes responsible for synthesizing secondary metabolites that scavenge ROS and exhibit antipathogenic and antipest properties, as well as genes encoding various PR proteins and antioxidant enzymes. In addition, the upregulated genes were associated with the synthesis of auxin, JA, and ABA, as well as the activation of transcription factors responsible for regulating plant growth and stress tolerance.

## 2. Materials and Methods

### 2.1. Isolation, Identification and Characterization of BcD1

The bacterial colony (designed as BcD1) was isolated by depositing 100 µL of compost suspension on nutrient agar (NA) containing 0.5% (*w*/*v*) peptone, 0.5% (*w*/*v*) NaCl, 0.3% (*w*/*v*) yeast extract, and 1.5% (*w*/*v*) agar. The genomic DNA of strain D1 was purified using the method described by Griffiths et al. [43]. DNA fragments of 16S rDNA were obtained by PCR amplification using primers fD1 (5′AGAGTTTGATCCTGGCTCAG3′) and rP1 (5′ACGGTTACCTTGTTACGACTT3′) [44]. The 16S rDNA PCR fragment was sequenced using a 3730 DNA Analyzer (Applied Biosystems^®^; Foster City, CA, USA). Obtained 16S rDNA sequences were analyzed using the Basic Local Alignment Search Tool (BLAST) program [45]. A phylogenetic tree was constructed by ClustalX2.1 and MEGA-X software [46,47].

#### 2.1.1. De Novo Whole Genome Assembly

The genome DNA of BcD1 was fragmented using the Celero PCR workflow with an Enzymatic Fragmentation DNA-Seq Kit and sequenced using the paired-end method of the Illumina MiSeq system. The quality of the sequence raw data was analyzed using NanoPlot v1.28.1 [48]. Subsequently, the reads were trimmed by removing adapters, low-quality sequences (Q20), and ambiguous bases. SPAdes v.3.14.1 [49] was used to perform the de novo assembly of the genome sequencing data. The open reading frame (ORF) was predicted by GlimmerHMM [50]. Prediction of rRNA and tRNA by RNAmmer and tRNAscan SE, respectively [51,52]. Gene sequences were annotated using the NCBI database. Gene function analysis was performed using FastAnnotator [53] and the Gene Ontology Consortium (http://geneontology.org/ accessed on 31 March 2023). The phylogenetic classification of protein families was analyzed based on the cluster of orthologous groups (COG) database (http://www.ncbi.nlm.nih.gov/COG accessed on 31 March 2023).

#### 2.1.2. Antifungal Activity

The antimicrobial activity of BcD1 was determined by a dual culture assay for detecting nonvolatile compounds and the inverse double technique for detecting volatile antibiotic compounds, respectively [54,55]. In brief, in the dual culture assay, two pieces of filter papers containing 10 µL of H_2_O (as a control) and 10^8^ CFU/mL of the test bacterial isolate were placed 3 cm away from the mycelial plug of *Foc* TR4 in the potato dextrose agar (PDA) medium. For the inverse double assay, *Foc* TR4 and BcD1 were grown in separate Petri dishes on PDA medium. The plate with the fungus was then inverted and positioned on top of the plate with the bacterium, both without lids, and sealed with Parafilm M to avoid the escape of VOCs from the headspace of the bacteria and fungi. Co-cultures of *Foc* TR4 with the test bacterial isolated were incubated at 28 °C for 4 days and 7 days in the dual culture and inverse double assay, respectively. The mycelium growth inhibition rate (I) was calculated using formula: I = (1−T/C) × 100, in which C and T indicate the mycelium diameter of *Foc* TR4 co-cultured with H_2_O and BcD1, respectively. The mean and standard error of the mycelium inhibition rate were calculated from the results of three replicates.

#### 2.1.3. Protease Activity

The proteolytic activity of the bacterial strain was analyzed qualitatively by inoculating the bacterial strain in NA plates containing 5% skim milk and cultured for 24 h at 28 °C. Protease activity was confirmed by the appearance of a clear zone surrounding the bacterial colonies. To prepare for zymogram analysis, the crude protease extract from a 24-h bacterial culture was precipitated by adding ammonium sulfate to achieve 60% saturation. The resulting protein pellet was resuspended in 50 mM phosphate buffer pH 7.0 and then dialyzed against the same buffer at room temperature for 16 h using a membrane with a molecular weight cut-off of 12,000–14,000 Da. The purified protease was analyzed on a 10% polyacrylamide gel under non-reducing conditions. Following completion of gel electrophoresis, the gel was washed with a solution of 2.5% Triton X-100 and subsequently incubated with 1% casein dissolved in a 50 mM phosphate buffer pH 7.0 for 90 min at room temperature. Finally, the gel was stained with Coomassie brilliant blue, and the appearance of a clear zone on the gel indicated the protease activity that led to casein degradation.

#### 2.1.4. Quantitation of IAA and Phosphate Solubility

To measure IAA production, BcD1 was cultured in Luria Broth (LB) medium supplemented with 2 mM L-tryptophan for 48 h. Supernatants of bacterial culture were collected for IAA quantitation using the Salkowski reagent [56]. For the qualitative analysis of phosphate solubilizing activity, 10 µL of BcD1 suspension overnight cultured in the LB medium was spotted onto the Pikovskaya (PVK) agar plate [57] and incubated at 28 °C for 2 weeks. The appearance of a clear zone surrounding the bacterial colony was an indication of phosphate-solubilizing activity.

### 2.2. Plant Experiments

#### 2.2.1. Growth Promoting

The effect of the isolated bacterial strain on promoting plant growth was analyzed in *Arabidopsis thaliana* ecotype Columbia-0 (Col-0). Four-day-old *Arabidopsis* seedlings grown in the Murashige and Skoog (MS) medium were co-cultured with the bacterial inoculants (5 colonies of BcD1) for 7 days, and root development of the treated seedlings was analyzed, including the number of lateral roots and root hairs. The growth-promoting effect of the isolated bacterial strain was further analyzed in 2-week-old *Arabidopsis* seedlings grown in soil. The bacterial isolate cultured in medium containing 0.5% sucrose, 0.5% peptone, 0.5% MgSO_4_, 0.04% KH_2_PO_4_, 0.03% K_2_HPO_4_, and 0.5% yeast extract at 28 °C in the dark for 24 h was centrifuged with the Avanti J-30I centrifuge (Beckman Coulter) at 5000× *g* for 10 min. The resulting bacterial pellet was resuspended in water to a density of 1 × 10^8^ CFU/mL and this bacterial suspension was used for plant treatment via foliar spray. Bacterial treatment was performed once a week, and the chlorophyll content and fresh weight of the seedlings were recorded after three treatments. Chlorophyll content was determined as described by Kurniawan et al. [42].

#### 2.2.2. Lignin, Glucosinolate, Triterpene, Flavonoid, and Total Phenolic Content

*Arabidopsis* seedlings treated with the bacterial isolate were harvested for analysis of secondary metabolites. To quantify lignin content, 0.5 g of leaf tissues were extracted using 100 mM phosphate buffer pH 7.4 containing 0.5% Triton X-100. The resulting pellets were washed with methanol and resuspended in a solution containing 2 N HCl and thioglycolic acid (TGA). The lignin contents were determined by following the procedures described by Bruce and West [58]. To quantify glucosinolates content, 0.1 g of leaf tissue was extracted with 2 mL of 80% methanol. The glucosinolates content was then determined using the methods described by Mawlong et al. [59]. For triterpenoids quantitation, 0.1 g of leaf tissue was extracted using 1 mL of methanol. The resulting supernatants were analyzed to determine the total triterpenoids content following the procedures described by Chang et al. [60]. Triterpenoids concentration was calculated using a standard curve generated from known concentrations of ursolic acid, and the results were expressed as mg of ursolic acid equivalents (UE) per gram of extract. The quantification of flavonoid contents was conducted following the procedures described by Quettier-Deleu et al. [61]. The total flavonoids content was determined based on a standard curve constructed by rutin with known concentrations. The results were expressed as µg of rutin equivalents (RE)/gram of extract. To determine the total phenolic content (TPC), the leaf tissues were extracted using acetone and then added to the diluted Folin–Ciocalteu reagent, following the methods described by Li et al. [62]. The total phenolic contents were quantified based on a standard curve generated from gallic acid with known concentrations, and the results were expressed in mg gallic acid equivalents (GAE) per gram of extract. All analyses were conducted in triplicate.

#### 2.2.3. Antioxidant Activity

To measure the activity of antioxidant enzymes, including POD and catalase, 0.1 g leaf tissues harvested from *Arabidopsis* seedlings treated with the bacterial isolate were ground in liquid nitrogen and extracted with 0.2 M potassium phosphate buffer containing 0.1 mM EDTA, pH 7.8. POD and catalase activity were analyzed in the resulting supernatants following the methods described by Aebi and Lester [63]. For the measurement of radical scavenging activity, 0.1 g of leaf tissues were ground in 80% methanol, and the resulting supernatants were mixed with 0.5 mM 2,2-diphenyl-1-picrylhydrazyl (DPPH) and 100 mM acetate buffer (pH 5.5). After incubating the mixture in the dark for 30 min, the absorbance at 517 nm was measured. Distilled water was used as a control. The free radical scavenging activity (%) was calculated using the following formula: [(A0—A1)/A0 × 100], where A0 is the absorbance of the control and A1 is the absorbance of the sample. Leaf tissues were ground in 80% ethanol, and the resulting supernatants were used for quantification of H_2_O_2_ concentration using the ferrous oxidation-xylenol orange (FOX) assay method [64]. All experiments were conducted in triplicate.

#### 2.2.4. Effect on Stress Tolerance

To perform the heat stress analysis, 2-week-old *Arabidopsis* seedlings, pretreated with the bacterial isolate at a concentration of 1 × 10^8^ CFU/mL, along with untreated seedlings used as a control, were exposed to a temperature of 45 °C for 45 min. After this, the heat-stressed seedlings were returned to 23 °C for 24 h, and the surviving plants were identified by the absence of wilted leaves. Survival rates were calculated by dividing the number of surviving seedlings by the total number of seedlings. Subsequently, the seedlings were grown at 23 °C for 7 days, and fresh weights were recorded at the end of the cultivation period. For the biotic stress analysis, 3-week-old *Arabidopsis* seedlings, pre-treated with the bacterial isolate, were sprayed with a soft rot pathogen, *Erwinia chrysanthemi* (1 × 10^4^ CFU/mL), and kept at room temperature for 24 h. Fifteen seedlings were included in each treatment, and the infected seedlings were identified by the presence of water-soaked tissues. To calculate the disease incidence rates, the number of infected seedlings was divided by the total number of seedlings. The experiments were conducted three times.

#### 2.2.5. RNA-seq Analysis

The leaf tissues from 2-week-old *Arabidopsis* seedlings treated with BcD1 were harvested for total RNA extraction using the method described Parcy et al. [65]. cDNA synthesized from the polyA-plus RNA purified from 5 μg of total RNA was used to construct the RNA-seq library by following Illumina’s protocols. After sequencing the library using the Illumina NextSeq 500 platform, the resulting sequences were analyzed following procedures described by Sukkasem, et al. [41]. FPKM (Fragments Per Kilobase of exons per Million mapped reads) values were used to quantify gene expression levels. The fold-change (FC) in gene expression was determined by dividing the expression levels of treatment by those of the control. Genes with FC values greater than 2.0 were regarded as up-regulated genes. The RNA-seq analysis was performed in two replicates using *Arabidopsis* samples isolated from two independent experiments.

#### 2.2.6. qPCR Analysis

One μg of total RNA was used for the synthesis of cDNAs using ImProm-II™ reverse transcriptase (Promega, Madison, WI, USA). The obtained cDNA was subjected to qPCR amplification using SYBR Green Master Mix. Relative fold changes in gene expression were analyzed using the 2^−ΔΔ^CT method in the StepOneTM Real-Time PCR System (Thermo Fisher, Waltham, MA, USA). The gene expression of *Actin 2* was analyzed as the reference gene for normalization. The primer sequences specific to the genes analyzed in this study were listed in Table 1.

### 2.3. Statistics

Treatment means were compared with SAS statistical software (version 3.8) using ANOVA and Tukey’s test. A *p*-value less than 0.05 indicated a statistically significant difference. Data were presented in mean  ±  SD of three replicates.

## 3. Results and Discussion

### 3.1. Isolation, Identification and Characterization of BcD1

**Figure 1:**
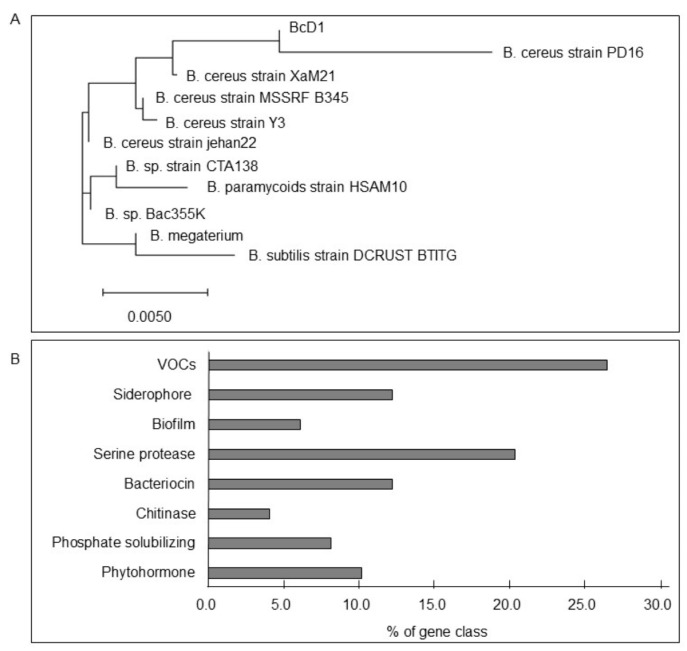
Genomic features of BcD1. (**A**) A phylogenetic tree constructed based on the 16S rDNA sequences. (**B**) The classification of genes identified in the BcD1 genome responsible for the synthesis of plant growth-promoting metabolites.

The analysis of the 16S rDNA sequence of rhizobacterial strain D1 using the BLASTN tool of NCBI showed a 96–97% match with several bacterial strains of the *Bacillus* species. A phylogenetic tree constructed using the neighbor-joining method in ClustalX2.1 and MEGA-X software showed that rhizobacterial strain D1 was grouped in the same clade as *Bacillus cereus* strain PD16. Hence, this newly isolated bacterial strain was designated as BcD1 (Figure 1A).

#### 3.1.1. De Novo Whole-Genome Assembly

A total of 2,979,681 reads were obtained and assembled to four contigs with a total genome size of 5.45 Mb and a GC content of 35.4%. Three plasmids ranged in size from 3239 bp to 275,984 bp. In total, 5489 open reading frames (ORFs) were predicted in the BcD1 genome; among them, 4983 (90.8%) ORFs matched the sequence of *B. cereus* and 484 (8.8%) ORFs matched other *Bacillus* species in the database. The identity of BcD1 obtained from the phylogenetic tree analysis was confirmed by the results of the genome sequence of BsD1. The BcD1 genome analysis revealed genes involved in the synthesis of various bioactive metabolites, such as VOCs, siderophore, biofilm components, serine proteases, bacteriocin, chitinase, phytohormones and phosphatases for phosphate solubilizing (Figure 1B). The detailed gene information was listed in Table 2. Genes involved in the synthesis of VOCs were identified in the BcD1 genome, which included six genes for producing 2,3-butanediol, four genes for synthesizing dimethyl disulfide, and three genes for generating terpenoids. Additionally, the BcD1genome contained six genes for synthesis of bacillibactin, a catechol-type siderophore, and three genes for synthesis of spermidine, a polyamine, which is a component of biofilm [66,67]. Microbial metabolites, including VOCs, siderophores, and biofilm components, have multifaceted functions in plant growth by suppressing pathogen growth and activating plant defense response [7,68,69]. Ten genes were identified in the BcD1 genome that encode two types of serine proteases: subtilisin-like serine proteases (subtilases) and trypsin-like serine proteases. Additionally, six genes were responsible for synthesizing bacteriocin, and two genes encoded chitinases. The antimicrobial and antipest properties of these bioactive compounds, including serine protease, bacteriocin, and chitinase, have been reported in previous studies [20,70,71,72]. The BcD1 genome contained four genes encoding acid phosphatase and alkaline phosphatase. These enzymes are involved in solubilizing phosphate complexes and improving phosphate availability in the soil [73]. The genome of BcD1 was found to have four genes responsible for IAA production and one gene responsible for synthesizing cytokinin hormone. IAA, together with cytokinin, plays an essential role in regulating plant growth and development by stimulating root development and improving the availability of nutrients [74]. In this study, a genome-wide analysis of BcD1 was conducted, which identified gene clusters for the synthesis of metabolites with diverse bioactivity associated with promoting plant growth through different mechanisms, such as pathogen suppression, induced defense response, and manipulation of root growth and nutrient availability. The two largest groups of genes were those involved in the synthesis of VOCs and serine proteases, which function in controlling pathogens and inducing stress tolerance.

#### 3.1.2. Antifungal Activity

**Figure 2:**
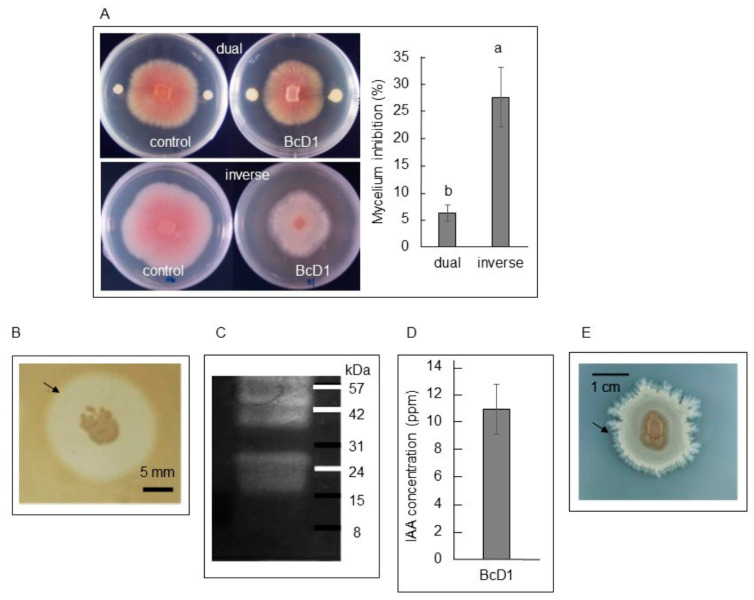
Characterization of BcD1. (**A**) Antifungal activity against *Fusarium oxysporum* f.sp. *cubense* Tropical Race 4 of diffusible metabolites in the dual culture assay (dual) and VOCs activity in the inverse double culture (inverse). (**B**) Production of extracellular protease on the Nutrient Agar medium containing skim milk. The clear zone surrounding the bacterial colony (arrow) indicated protease activity. (**C**) Zymogram assay to detect extracellular protease on native polyacrylamide gel electrophoresis and casein as a substrate. (**D**) IAA production. (**E**) Phosphate solubilizing activity on the PVK medium evidenced as the appearance of a clear zone (arrow) surrounding the colony. Values in each histogram are the mean of three replicates ± SD. In histogram A, different letters indicate statistical significance at *p* = 0.05.

As shown in Figure 2A, the BcD1 inoculant suppressed the mycelial growth of *Foc* TR4, the pathogen responsible for banana *Fusarium* wilt, in both the dual culture and inverse double assays. The results suggest that BcD1 produced both diffusible and volatile antifungal compounds. The volatile antifungal metabolites exhibited a stronger effect in inhibiting the mycelial growth of *Foc* TR4. This finding indicates that BcD1 has the ability to produce a considerable quantity of VOCs that exhibit significant bioactivity in inhibiting the growth of fungi.

#### 3.1.3. Protease Activity

The second largest group of genes associated with plant growth in the BcD1 genome was responsible for the synthesis of extracellular proteases. Inoculating BcD1 on a skim milk-containing medium resulted in the formation of a clear zone around the bacterial colony, which indicates the presence of extracellular protease activity (Figure 2B). Additionally, zymogram gel analysis confirmed the protease activity of BcD1, revealing the presence of three extracellular proteases between the molecular markers of 24 to 56 kDa (Figure 2C). The findings from this analysis indicate that BcD1 has the capacity to produce proteases outside of the cell. Previous research has reported that the *B. cereus* strain NJSZ-13 produces a 28 kDa extracellular alkaline protease, which acts as a pathogenicity factor and exhibits nematicidal properties [75].

#### 3.1.4. IAA Production and Phosphate Solubility

Many strains of PGPR were reported to produce various concentrations of IAA, which can affect plant growth [76]. Four genes related to the production of IAA were recognized in the genome of BcD1. This was supported by the detection of 11.40 ± 1.32 mg/L of IAA in the BcD1 culture (Figure 2D). This IAA concentration belongs to the low range of IAA concentrations produced by *Bacillus* isolates [77]. Likewise, BcD1’s genome had four genes linked to phosphate solubilization, and this was demonstrated by the capacity of the BcD1 culture to solubilize tricalcium phosphate in the PVK medium, as indicated by the formation of a small clear zone around the bacterial colony (Figure 1E). It is well known that rhizobacteria-produced IAA acts as a diffusible factor for changing lateral root development in host plants [78].

### 3.2. Plant Experiments

#### 3.2.1. Growth Promoting

**Figure 3:**
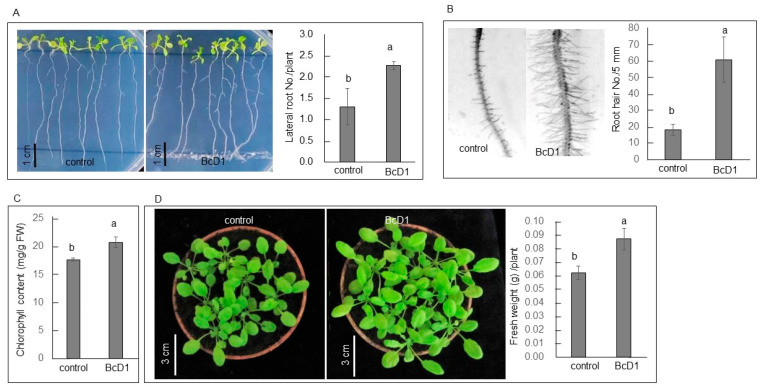
BcD1 treatment promoted *Arabidopsis* growth. Lateral roots (**A**) and root hairs (**B**) formation in seedlings cocultured with BcD1 for seven days. Chlorophyll content (**C**), plant stature, and fresh weight (**D**) of soil-grown seedlings treated once a week for three consecutive weeks with 1 × 10^8^ CFU/mL of BcD1. Values in histograms are the mean of three replicates ± SD. In each histogram, different letters indicate statistical significance at *p* = 0.05.

The *Arabidopsis* seedlings cocultured with BcD1 for seven days exhibited a greater number of lateral roots and root hairs (Figure 3A,B). Additionally, four-week-old soil-grown *Arabidopsis* seedlings treated with BcD1 displayed an increase in chlorophyll content in the leaf tissues (Figure 3C). The treated seedlings exhibited larger plant sizes than the control group, showing a 40% increase in fresh weight (Figure 3D).

The regulation of lateral roots and root hairs development in plants is associated with IAA [79,80]. BcD1 possesses genes responsible for IAA synthesis and produces a measurable quantity of IAA, which could potentially induce the development of lateral roots and root hairs in *Arabidopsis* seedlings. In addition to IAA, VOCs produced by rhizobacteria also influence root development by altering auxin homeostasis and perception in host plants. For example, the VOC metabolite dimethyl disulfide has been shown to manipulate the auxin signaling pathway, thereby altering root growth [81]. A prior investigation has also noted that the release of VOCs by *B. cereus* has been linked to an increase in sulfur absorption in plants colonized by the bacterium, ultimately resulting in enhanced plant growth [82]. Therefore, the observed changes in root structure and increased growth of seedlings could be attributed to the function of both VOCs and IAA produced by BcD1.

Since adding boxes to Figure 1 does not improve the quality, Figure 1 will remain without boxes.

#### 3.2.2. Lignin, Glucosinolate, Triterpene, Flavonoid, and Total Phenolic Content

**Figure 4:**
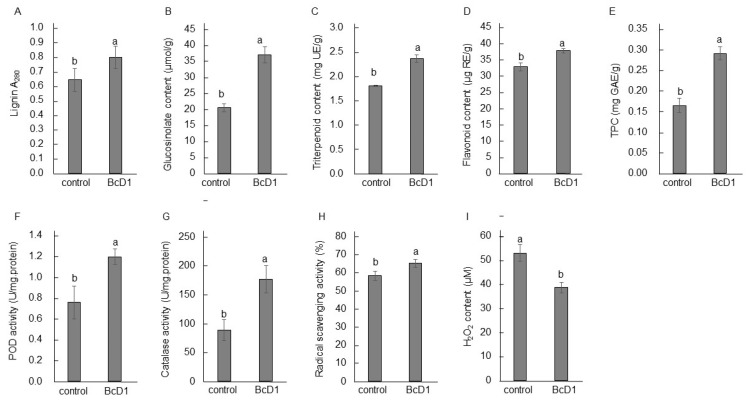
Biochemical features of *Arabidopsis* seedlings affected by BcD1 treatment. Soil-grown *Arabidopsis* seedlings were treated with BcD1 culture at a density of 1 × 10^8^ CFU/mL or water (control) once a week for three consecutive weeks. Concentration of lignin (**A**), glucosinolate (**B**), triterpenoid (**C**), flavonoid (**D**), and total phenolic compounds (TPC) (**E**). Activity of POD (**F**), catalase (**G**), DPPH radical scavenging (**H**), and H_2_O_2_ accumulation (**I**). Values in histograms are the mean of three replicate ± SD. In each histogram, different letters indicate statistical significance at *p* = 0.05.

BcD1 treatment resulted in a higher deposition of lignin in the *Arabidopsis* seedlings (Figure 4A). Increasing lignin accumulation in plants is a component of the induced immune response [83]. The application of BcD1 also stimulated the production of secondary metabolites, including glucosinolates, triterpenoids, flavonoids, and TPC, in the treated *Arabidopsis* seedlings (Figure 4B–E). The presence of glucosinolates and triterpenoids in plants is associated with their defense response to pathogen and pest attacks [25,26]. The majority of the antioxidant activity in plants can be attributed to flavonoids, which are a type of phenolic compound found in plant tissues [34].

#### 3.2.3. Antioxidant Activity

The application of BcD1 resulted in a 70% increase in POD and a 100% increase in catalase activity, as well as an enhancement in radical scavenging activity (Figure 4F–H). As a result, the treated seedlings exhibited a decrease of approximately 25% in H_2_O_2_ accumulation (Figure 4I). PGPR bacterial strains have been found to impact the secondary metabolites and antioxidant activity of their host plants [40,42]. The VOCs generated by PGPR have been observed to promote the synthesis of secondary metabolites and antioxidants, thereby enhancing plant growth under conditions of salt stress [16]. Rhizobacteria’s extracellular serine proteases are known to activate plant immunity and influence various cellular pathways, including the production of antimicrobial compounds and cell wall lignification in host plants [84]. These extracellular proteases also have a role in activating antioxidant enzymes in host plants, such as superoxide dismutase and polyphenol oxidase [85]. The activation of the antioxidant defense system, which includes enzymes and metabolites, by microbial VOCs and extracellular proteases, implies that BcD1 has the potential to prime the physiology of plants to combat oxidative stress. The increased accumulation of lignin and secondary metabolites associated with disease resistance demonstrates the effectiveness of BcD1’s VOCs and serine proteases in activating induced immunity in *Arabidopsis* seedlings.

#### 3.2.4. Effect on Stress Tolerance

**Figure 5:**
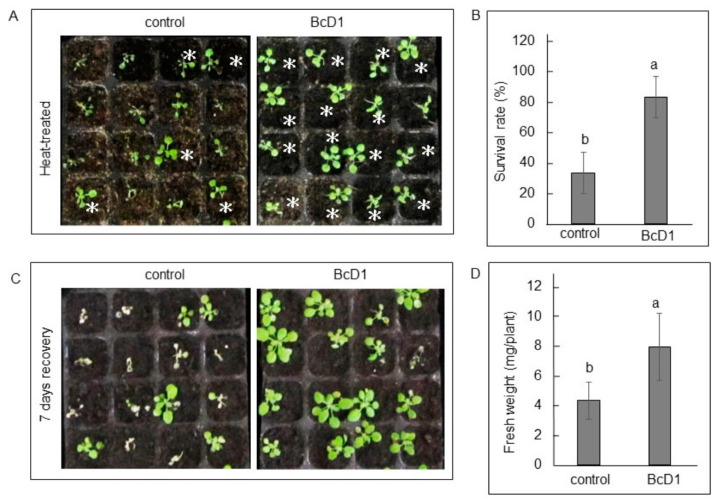
Heat stress tolerance induced by BcD1 treatment. Two-week-old *Arabidopsis* seedlings were foliar sprayed with BcD1 culture at a density of 1 × 10^8^ CFU/mL, or left untreated (control). The seedlings were then exposed to 45 °C for 45 min and allowed to recover at 23 °C for 24 h (**A**). The survival rates of the heat-stressed seedlings were calculated by dividing the number of unwilted seedlings by the total number of seedlings (**B**). The heat-stressed seedlings were incubated at 23 °C for seven days (**C**), and their fresh weights were measured (**D**). Asterisks (*) indicated unwilted seedlings. Values in histograms are the mean of three replicate ± SD. In each histogram, different letters indicate statistical significance at *p* = 0.05.

The BcD1-pretreated seedlings exhibited a lower number of wilted plants after exposure to heat stress (Figure 5A). Pretreatment of BcD1 increased survival rate with approximately 50% (Figure 5B). Following a seven-day recovery period, the seedlings treated with BcD1 displayed a significant increase in size, with their fresh weight being approximately 80% greater than that of the untreated seedlings (Figure 5C,D).

The exposure of plants to heat stress causes the accumulation of ROS, which can result in increased oxidative stress and ultimately lead to programmed cell death (PCD) [86]. Previous studies have shown that flavonoid content is linked to heat stress tolerance, as they offer antioxidant activity [35,87]. Glucosinolates and terpenoids are known for their significant role in combating biotic stress; however, exogenous application of glucosinolates has been demonstrated to enhance heat stress tolerance [88]. Moreover, a lack of glucosinolate synthesis leads to a thermosensitive phenotype in *Arabidopsis* [89]. The increased synthesis of terpenoids helps to reduce ROS under heat stress condition [90]. Hence, the increased content of flavonoids, glucosinolates, and terpenoids, responding to BcD1 treatment, along with the increased activity of antioxidant enzyme, may contribute to the enhanced heat stress tolerance in *Arabidopsis* seedlings.

**Figure 6:**
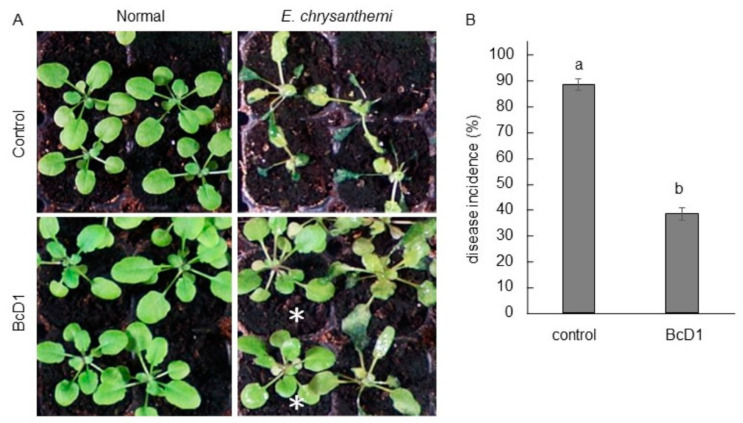
Disease resistance induced by BcD1 treatment. Three-week-old *Arabidopsis* seedlings were either foliar sprayed with BcD1 at a density of 1 × 10^8^ CFU/mL or left untreated as a control. Both the BcD1-treated and control seedlings were inoculated with *Erwinia chrysanthemi*. After 24 h of inoculation, some seedlings remained healthy and were marked with asterisks (*), while some seedlings showed water-soaked symptoms indicating infection by the pathogen (**A**). The percentage of disease incidence was calculated by dividing the number of infected seedlings by the total number of seedlings (**B**). Values in histograms are the mean of three replicate ± SD. In each histogram, different letters indicate statistical significance at *p* = 0.05.

*Arabidopsis* seedlings without BcD1 pretreatment displayed evident disease symptoms, such as water-soaked tissues, whereas the BcD1-pretreated seedlings showed less severe disease symptoms (Figure 6A) and a disease incidence that was approximately 50% lower than that of the control seedlings (Figure 6B).

Several studies have demonstrated that strengthening cell wall structure by increasing lignin deposition can improve disease resistance against bacterial soft rot [91,92,93]. VOCs produced by rhizobacteria, such as dimethyl disulfide and 2,3-butanediol, are demonstrated to induce systemic resistance against pathogens in host plants [14,15]. Alternatively, extracellular serine proteases from rhizobacteria activate plant immunity and impact cell wall lignification and antimicrobial compound production in host plants [84]. Glucosinolates and phenolic compounds have been correlated with induced resistance against bacterial soft rot [94,95,96,97]. The findings of this study propose that the VOCs and serine protease generated by BcD1 can effectively manage bacterial soft rot disease by promoting the production of lignin and secondary metabolites such as glucosinolates and phenolic compounds.

#### 3.2.5. RNA-seq Analysis

**Figure 7:**
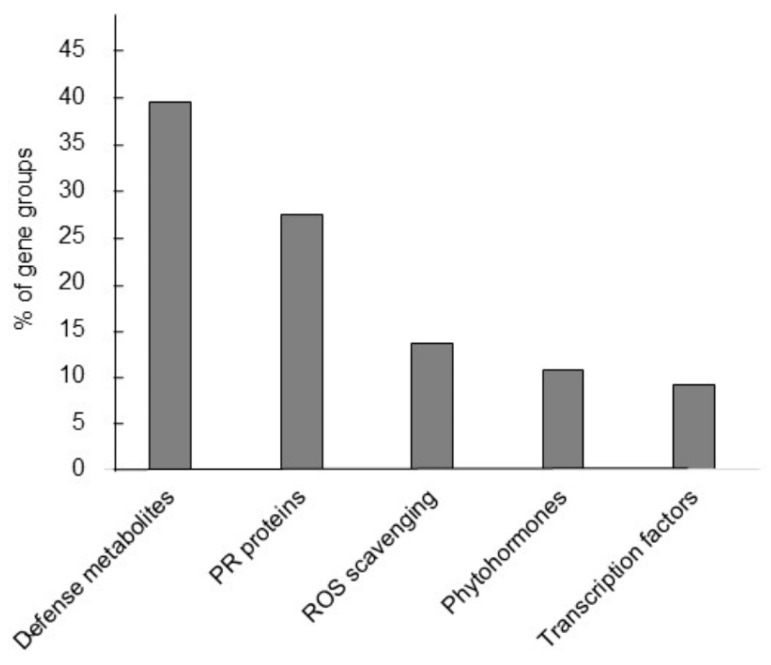
Stress related genes identified in *Arabidopsis* transcriptome treated with BcD1. Upregulated genes with an induction fold change greater than 2.0 were classified into five groups based on their annotated functions.

The results of transcriptome analysis revealed 66 upregulated genes with assigned functions linked to cellular pathways that regulate stress tolerance. These genes were grouped into five categories based on their roles in producing defense metabolites, PR proteins, ROS scavenging products, phytohormones, and transcription factors for stress response (Figure 7). As shown in Table 3, BcD1 upregulated seven members of *berberine bridge enzyme (BBE)-like* and *caffeoyl-CoA O-methyltransferase* (*CCOAMT*), both of which are involved in the synthesis of lignin [98,99]. The upregulated genes identified in this study were involved in the synthesis of terpenoids, such as *terpene synthase 04* (*TPS4*), *MARNERAL SYNTHASE 1* (*MRN1*), and *thalianol synthase* (*AtTHAS1*) [100,101,102]. BcD1 treatment increase expression of genes, including *CYP71A12* and *senescence-associated protein 13* (*SAG13*), which are participated in the synthesis of alkaloid [103,104]. Four members of *INDOLE GLUCOSINOLATE O-METHYLTRANSFERASE* (*IGMT*) and *CYP81F2* implicated in the synthesis of glucosinolates [105] were identified in this study. The expression of *CYP82C2* was increased by BcD1 treatment. This gene functions in the production of 4-hydroxy indole-3-carbonyl nitrile (4-OH-ICN), a cyanogenic phytoalexin in *Arabidopsis* [106]. BcD1 also induced the expression of genes involved in the production of osmolytes, in which *trehalose synthase 11* (*ATTPS11*) for trehalose synthesis and *delta1-pyrroline-5-carboxylate synthase 1* (*P5CS1*) for proline synthesis [107,108]. Several upregulated genes were members of *GDSL lipases*. The expression of *GDSL* members is linked to generate of lipid signal for induction of systemic resistance against bacterial soft rot and green peach aphids [109,110]. Two genes associated with antipest function were upregulated, such as *CYP81D11*, transcriptionally linked to defense response to insect damage [111], and *NATA1* for synthesis of N(δ)-acetylornithine, associated with resistance against green peach aphids [112]. The results of this study show that BcD1 is a potential elicitor for synthesis defensive metabolites, including lignin, terpenoids, alkaloids, glucosinolates, cynogenic compound, osmolytes, lipid signal molecules, and antipest metabolites (Table 3). All of the upregulated genes that respond to BcD1, except for those involved in producing osmolytes, are responsible for synthesizing defense metabolites that can induce resistance against pathogens and insects.

Upregulated genes included five members of genes encoding protease inhibitors (PR-6), which were serine protease inhibitor (SPI), kunitz family trypsin and protease inhibitor (ATKTI5), kunitz trypsin inhibitor 1 (KTI1), TRYPSIN INHIBITOR PROTEIN 1 (TI1), and UNUSUAL SERINE PROTEASE INHIBITOR (UPI) (Table 3). Serine protease/trypsin inhibitors play an important role in plant defense against pests and pathogens [113]. The second group was Defensins/PDF gene family (PR-12), known to play a significant role in regulating plant disease resistance; however, its role in controlling plant abiotic stress tolerance is also reported [114,115]. Overexpression of a defensin gene resulted in enhanced heat stress tolerance in Arabidopsis [116]. BcD1 upregulated four members of chitinases (PR-3) and two members of PR-2, both of which play a role in immunity triggered by microbial molecules [117,118]. Antimicrobial propertied of THIONIN 2.1 (THI2.1; PR-13) and osmotin 34 (OSM34; PR5) have been demonstrated [119,120]. Additionally, OSM34 plays a role in sensing the ABA signal [121]. The results of this study show that several PR genes, including PR-6, PR-12, PR-3, and PR-13, induced by BcD1 treatment contribute to induced disease resistance. However, PR-12 and PR-5 have dual function that relates to both biotic and abiotic stress tolerance.

In response to BcD1 treatment, a significant number of genes showing functions of reducing oxidative stress were increased in expression. As shown in Table 3, BcD1 upregulated genes encoding a tau class of glutathione transferases (GSTU10), two members of methionine sulfoxide reductase (MSRB), a glyoxalase (GLY), and five members of peroxidases (PRX). GST is a class of detoxification enzymes for oxidative stress [122]. MSRB is a stress-related peroxidase [123]. GLY is involved in the detoxification of methylglyoxal oxidative stress [124]. PRX71 catalyzes the lignification in the cell walls [125]. Peroxiredoxin-2C (PRXIIC), a thiol peroxidase, can detoxify peroxides during oxidative stress [126].

Plant hormones play essential roles in controlling diverse aspects of plant growth, development, and stress tolerance [127]. BcD1 treatment increased the expression of *NITRILASE 2* (*NIT2*) (Table 3). This gene is involved in synthesis of IAA via tryptophan dependent pathway [128]. Similar to previous study, the VOC metabolite of 2,3-butanediol produced by *Bacillus* strain has been shown to activate the expression of gene encoding nitrilases [129]. BcD1 induced expression of the *SHORT-CHAIN DEHYDROGENASE REDUCTASE 4* (*SDR4*), whose gene product is involved in the ABA biosynthesis pathway [130]. Spermidine-producing rhizobacteria were able to increase the ABA content in the colonized plants [7]. Correspondingly, the genome of BcD1 contains genes related to the synthesis of spermidine. Two upregulated genes encoding lipoxygenase (LOX) and alpha-dioxygenase (DOX1) responsible for the conversion of cis-(+)-12-oxo-phytodienoic acid (OPDA) to JA [131]. Additionally, the upregulated gene encoding SABATH methyltransferase (BSMT1), is involved in the synthesis of methyl salicylate (MeSA), affecting emission of SA [132]. Previously, rhizobacteria that produced dimethyl disulfide were able to activate the SA signaling pathway to improve plant disease resistance in the past. In contrast, those producing 2,3-butanediol triggered a plant defense response by activating the SA, JA, and ethylene signaling pathways [14,15,133]. Moreover, genes involved in the GA biosynthesis pathway, such as *GA3OX1* and *GA20OX2* [134], were found to be induced expression in the transcriptome analyses. Thus, BcD1 treatment altered hormone homeostasis including IAA, ABA, JA, and GA. Among these, IAA and GA are growth-promoting hormones, whereas ABA and JA are hormones involved in regulating stress tolerance [135]. Moreover, VOCs and spermidine produced by BcD1 might be bioactive compounds for inducing phytohormone signals regulating stress tolerance. BcD1 treatment upregulated a large number of genes for synthesis of metabolites showing functions associated with disease resistance and resolving oxidative stress. The JA signal plays a strong role in the signaling pathway regulates insect resistance [136]. These results suggest that the JA signaling pathway plays a stronger role in inducing stress tolerance in *Arabidopsis*.

BcD1 treatment resulted in the upregulation of a group of transcription factors known to be involved in regulating both abiotic and biotic stress response (Table 3). *WRKY30* and *WRKY71* are associated with abiotic and biotic stress response [137,138,139,140]. In addition, *WRKY61* were found to regulate plant immunity toward viral infection [141], while the expression of *WRKY31* was induced under cadmium stress in pak choi [142]. The expression of *SRG3*, which belongs to the *S-nitrosothiol (SNO) regulated gene* (*SRG*) family targeted by nitric oxide (NO) during plant immunity, was induced by BcD1 treatment. *MYB54* regulates expression of *secondary wall-associated NAC domain protein1* (*SND1*) for synthesis of secondary cell wall [143].

The transcriptome analysis reveals a large proportion of BcD1 upregulated genes associated with synthesis metabolites functioning in cellular pathway linked to disease resistance. BcD1 also increased the expression of *PR* genes, mostly linked to the antipest property. Serine proteases produced by BcD1 elicit the expression of genes encoding serine protease inhibitors in *Arabidopsis* seedlings. Plant protease inhibitors are promising biocontrol agents for pest management [144]. BcD1 treatment induced the expression genes involved in the synthesis of IAA, JA, and ABA, which may regulate transcription factors associated with plant growth and stress response.

#### 3.2.6. qPCR Analysis

**Figure 8:**
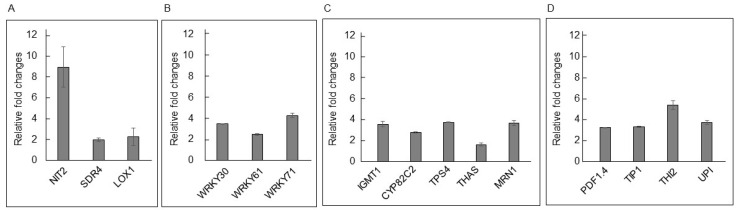
qPCR analysis of *Arabidopsis* genes activated by BcD1 treatment. The qPCR technique was used to measure the fold changes in gene expression of genes identified from a transcriptome study. (**A**) Genes were involved in synthesis of phytohormones. (**B**) Genes encoded transcription factors. (**C**) Genes were involved in synthesis of secondary metabolites. (**D**) Genes encoded PR proteins. NIT2: nitrilase 2, SDR4: SHORT-CHAIN DEHYDROGENASE REDUCTASE 4, LOX1: LIPOXYGENASE 1, WRKY30: WRKY DNA-binding protein 30, WRKY61: WRKY DNA-binding protein 61, WRKY71: WRKY DNA-binding protein 71, IGMT1: INDOLE GLUCOSINOLATE O-METHYLTRANSFERASE, CYP82C2: cytochrome P450, TPS4: terpene synthase 04, THAS: thalianol synthase, MRN1: MARNERAL SYNTHASE 1, PDF1.4: plant defensin 1.4, TIP1: TRYPSIN INHIBITOR PROTEIN 1, THI2: THIONIN 2, UPI: UNUSUAL SERINE PROTEASE INHIBITOR.

By analyzing qPCR data, BcD1 treatment was shown to activate the signaling pathways of auxin, ABA, and JA through the increased expression of *NIT2* for IAA synthesis, *SDR4* for ABA synthesis, and *LOX1* for JA synthesis (Figure 8A). As shown in Figure 8D, qPCR analysis confirmed the expression levels of three WRKY transcription factors, *WRKY30*, *WRKY61*, and *WRKY71*, which have roles in transcriptional regulation associated with acquiring tolerance to various stresses. Moreover, it was confirmed that BcD1 stimulated the expression of genes responsible for producing defense metabolites, including *IGMT1* for glucosinolate synthesis, *CYP82C2* for cyanogenic compound synthesis, and *TPS4*, *THAS*, and *MRN1* for terpenoid synthesis (Figure 8C). BcD1 treatment also triggered the expression of *PR* genes including *PDF1.4*, *TIP1*, *THI2*, and *UPI* (Figure 8D).

In this study, RNA-seq and qPCR methods were used to identify the signaling molecules affected by BcD1 treatment, including auxin, JA, and ABA. The results showed that the activated auxin signal was associated with altered root architecture and enhanced plant growth induced by BcD1 treatment. The increased JA signal was possibly related to the stimulation of corresponding transcription factors, leading to enhanced synthesis of lignin and metabolites of pathogen and antipest, as well as increased activity of antioxidant enzymes. These effects might contribute to the phenotypes of enhanced tolerance to heat stress and bacterial soft rot. The activated ABA signal may also play a role in the acquisition of heat stress tolerance in BcD1-treated seedlings.

## 4. Conclusions

The findings of this study indicate that BcD1 metabolites, such as VOCs and IAA, effectively enhance plant growth by modifying the root architecture and increasing nutrient absorption. Additionally, the VOCs and extracellular proteases from BcD1 work cooperatively to activate the JA signal, leading to increased lignin deposition, elevated production of secondary metabolites, and boosted activity of antioxidant enzymes, thereby strengthening plant resistance to heat stress and bacterial soft rot. The genes responsible for the synthesis of spermidine in BcD1 may be linked to the activated ABA signaling in *Arabidopsis* seedlings, potentially contributing to the acquisition of thermotolerance. It is worth noting that BcD1 produces a substantial quantity of extracellular proteases that can trigger the production of protease inhibitors and antipest peptides in plants, making it a promising option for controlling insect-borne diseases.

## Figures and Tables

**Table 1:** Sequences of primer used in qPCR analysis.

Gene	Forward Primer	Reverse Primer
*Actin 2*	5′CGGTAACATTGTGCTCAGTG3′	5′GTGAACGATTCCTGGACCTG3′
*IGMT1*	5′ACAATCGCGGTCGTTAAGAA3′	5′GCTCCACACCGGGATAAGAA3′
*CYP82C2*	5′CAGGCTCTTCATTGGCCATGC3′	5′CTTCCTTAAGACGTGGACTG3′
*TPS4*	5′GCCACTGATGGCACATGGTG3′	5′GTAGAAGCATGGTGCGAATA3′
*THAS1*	5′GAAGCAATTCGTAAAGCAGT3′	5′GAGACGTCGCAGAGCATGTG3′
*MRN1*	5′TCTGAAGCTATACGTAGAGC3′	5′CGCAGAGCATTTGTGTACAA3′
*PDF1.4*	5′ATGGCTTCTTCTTACACACT3′	5′AGCAGAAACATGCGAAACCC3′
*TIP1*	5′ATGGCAAAGGCTATCGTTTC3′	5′GTTACTGCCCTGTCCCCAAC3′
*THI2*	5′CTGCCCTTCCAACCAAGCTA3′	5′TTGTTCCGACGCTCCATTCA3′
*UPI*	5′AAAGCTCATGGCCAGAGCTT3′	5′CGATGATAGGAATTTGAACA3′
*NIT2*	5′CGTTTACGACACTCCGATTG3′	5′CTGGTCTCGAGTAATGTCCA3′
*SDR4*	5′GCTTCTAAGCACGCGCTTCT3′	5′TCATGAGCTTAACGACGCTA3′
*LOX1*	5′CGGACAGTATCCAGTTGCTG3′	5′GTTCTTGAGAGTGTCGTCGT3′
*WRKY30*	5′GATAGAACGCTGGACGATGG3′	5′CGGTTCGAGGTTTTGTATCG3′
*WRKY61*	5′GTGCAGCTTACGGCAACATT3′	5′CAGCCGGTAAAGATGGCACT3′
*WRKY71*	5′CATCCGATCCCATCGACGTT3′	5′GAAGGAACAATGTCCTGAAG3

**Table 2:** Genes identified in the BcD1 genome linked to the synthesis of metabolites associated with plant growth promotion.

Acc. No.	Gene Description	Position
Bacteriocin		
EDX55115	bacteriocin O-metyltransferase	407364-407603
WP_001071385	heterocycloanthracin/sonorensin family bacteriocin	4222458-4222213
WP_041184522	heterocycloanthracin/sonorensin family bacteriocin	4290218-4289925
WP_014893786	bacteriocin-processing peptidase family protein	4709577-4705375
WP_046648645	bacteriocin biosynthesis protein SagD	58276-56987
WP_000067649	thiazole-containing bacteriocin maturation protein	364592-366403
Siderophore		
-bacillibactin		
WP_000616755	isochorismate synthase DhbC	2193083-2194477
WP_001133933	non-ribosomal peptide synthetase EntF	4559229-4552072
WP_001007250	isochorismatase DhbB	24560156-4559263
WP_000955359	(2,3-dihydroxybenzoyl)adenylate synthase EntE	4561797-4560181
WP_000657800	isochorismate synthase DhbC	4563009-4561810
WP_001048422	2,3-DHB DhbA	4563806-4563036
VOCs		
- 2,3-butanediol		
WP_000215033	alpha-acetolactate decarboxylase	721764-721006
WP_000813479	acetolactate synthase large subunit	5010240-5008525
WP_000822944	acetolactate synthase small subunit	247570-247061
WP_000095846	acetolactate synthase large subunit	249267-247567
WP_000642458	2,3-butanediol dehydrogenase	3763547-3764614
AAS39887	acetolactate synthase	723478-721781
-dimethyl disulfide		
WP_000460299	methionine gamma-lyase	3328355-3329626
WP_000726591	methionine gamma-lyase	2387529-2388707
WP_001201908	cystathionine gamma-lyase	2694257-2695390
WP_000122291	cystathionine beta-lyase	2797135-2795972
-terpenoids		
WP_000251030	IPP isomerase	153587-152538
EEL15746	DXP reductoisomerase	3201707-3202921
WP_000288295	MEP cytidylyltransferase, ispD	1488120-1487440
Phytohormones		
-IAA		
WP_000080294	aldehyde dehydrogenase DhaS	3490691-3489207
WP_000537830	tryptophan synthase trpA	380153-379377
WP_001105023	tryptophan synthase trpB	381350-380157
WP_000536712	tryptophan synthase trpC	382719-381958
-Zeatin		
WP_000504938	MiaA	3319276-3320229
Biofilm		
-Spermidine		
WP_000424696	spermidine synthase	1716349-1717176
EEK42871	S-adenosylmethionine decarboxylase	2459113-2459430
WP_001209831	agmatinase	1717394-1718266
Chitinase		
WP_000837164	chitinase	1149777-1151819
WP_000932466	chitinase	3309435-3308353
Serine protease		
WP_001089044	serine protease	4854324-4853374
WP_000747582	serine protease	335901-336728
WP_000008058	serine protease	1616343-1617518
WP_000728874	subtilisin-like serine proteases	2178374-2174151
WP_000754169	subtilisin-like serine proteases	2711340-2708572
WP_000689206	subtilisin-like serine proteases	3274577-3276418
WP_000820235	subtilisin-like serine proteases	4423951-4419710
WP_000790939	subtilisin-like serine proteases	4545913-4547106
WP_000542636	trypsin-like serine proteases	3435940-3437181
WP_041184482	trypsin-like serine proteases	1930012-1931196
Phosphate solubilizing		
WP_080120806	metallophosphoesterase	768312-765853
WP_000356445	phosphodiesterases	1142793-1144106
WP_000714924	alkaline phosphatase, PhoA	2717644-2719029
WP_000067230	alkaline phosphatase, PhoA	3951628-3953313

**Table 3:** Upregulated genes associated with stress response.

acc. No.	Gene	FC1	FC2	acc. No.	Gene	FC1	FC2
defense metabolites							
-lignin							
AT1G30700	ATBBE8	2.4	4.4	AT1G30720	ATBBE10	4.1	5.6
AT1G30730	ATBBE11	4.2	5.4	AT1G26420	ATBBE7	2.1	3.4
AT1G26380	ATBBE3	2.2	5.0	AT1G26390	ATBBE4	5.1	10.9
AT1G26410	ATBBE6	4.8	18.2	AT1G67980	CCOAMT	2.0	5.7
-terpenoids							
AT1G61120	TPS4	2.7	3.9	AT5G42600	MRN1	8.3	31.1
AT5G48010	THAS1	2.0	9.1				
-alkaloids							
AT2G30750	CYP71A12	3.0	4.4	AT2G29350	SAG13	4.6	6.6
-glucosinolates							
AT1G21100	IGMT1	2.2	2.4				
AT1G21110	IGMT3	2.8	2.8	AT1G21120	IGMT2	2.3	2.4
AT1G76790	IGMT5	2.0	2.2	AT5G57220	CYP81F2	2.9	2.3
- phytoalexin							
AT4G31970	CYP82C2	2.7	9.1				
-osmolytes							
AT2G18700	ATTPS11	2.2	1.7	AT2G39800	P5CS1	3.1	2.8
-lipid signal							
AT1G54020	GLIP	4.6	9.9	AT5G40990	GLIP1	4.6	27.5
-pest resistance							
AT5G14180	MPL1	2.2	3.9	AT3G28740	CYP81D11	5.2	4.3
AT2G39030	NATA1	4.0	4.9				
PR proteins							
-PR-6							
AT5G43570	PR-6	14.2	20.1	AT1G17860	ATKTI5	2.0	2.6
AT1G73260	KTI1	7.3	9.1	AT2G43510	TI1	3.8	6.7
AT5G43580	UPI	4.2	7.4				
-PR12							
AT3G59930	DEFL	3.8	3.0	AT5G44420	PDF1.2	6.0	4.6
AT5G44430	PDF1.2C	3.8	3.9	AT1G19610	PDF1.4	3.6	10.1
AT2G26010	PDF1.3	6.1	4.2				
-PR3							
AT5G24090	CHIA	2.0	3.2	AT2G43570	CHI	2.2	2.0
AT2G43620	CHI	4.0	2.2	AT2G43590	PR-3 like	2.9	2.0
-PR2							
AT3G57260	PR2	2.9	2.0	AT4G16260	PR2	2.4	2.0
-PR13							
AT1G72260	THI2.1	3.2	10.9				
-PR5							
AT4G11650	OSM34	5.2	5.9				
ROS scavenging							
AT1G74590	GSTU10	2.4	6.8	AT4G04810	MSRB4	2.0	3.5
AT4G21830	MSRB7	2.2	3.4	AT1G80160	GLY17	3.5	2.9
AT5G64120	PER71	2.6	3.1	AT2G18150	PER	2.2	2.0
AT5G19880	PER	6.6	31.3	AT3G49120	PERX34	2.0	2.2
AT1G65970	PRXIIC	2.1	3.4				
Phytohormones							
AT3G44300	NIT2	7.9	11.1	AT3G29250	SDR4	3.9	6.1
AT3G01420	DOX1	2.4	4.1	AT1G53903	LOX	2.9	2.7
AT3G11480	BSMT1	4.7	6.4	AT1G15550	GA3OX1	3.0	3.2
AT5G51810	GA20OX2	2.2	3.6				
Transcription factors							
AT3G02040	SRG3	2.0	2.0	AT5G24110	WRKY30	4.0	2.1
AT1G18860	WRKY61	3.9	5.3	AT4G22070	WRKY31	3.2	6.0
AT1G29860	WRKY71	3.3	5.7	AT1G73410	MYB54	2.8	4.3

FC1 and FC2: the fold change of gene expression obtained from two RNA-seq analyses.

## Data Availability

The data presented in this study are available in the article.
